# Applications of laser technology in the manipulation of human spermatozoa

**DOI:** 10.1186/s12958-023-01148-9

**Published:** 2023-10-21

**Authors:** Yamei Xue, Yuping Xiong, Xiaohong Cheng, Kun Li

**Affiliations:** 1 Assisted Reproduction Unit, Department of Obstetrics and Gynecology, Sir Run Run Shaw Hospital, School of Medicine, Zhejiang University, Hangzhou, China; 2 Institute for Reproductive Health, School of Pharmacy, Hangzhou Medical College, Hangzhou, China

**Keywords:** Infrared lasers, Photobiomodulation therapy, Laser optical trap, Laser-assisted selection, Laser-assisted immobilization, Sperm motility, Male infertility, Assisted reproductive technology (ART)

## Abstract

The application of laser technology in the field of assisted reproductive technology (ART) has experienced rapid growth over the past decades owing to revolutionary techniques such as intracytoplasmic sperm injection (ICSI), preimplantation genetic testing (PGT), and in vitro manipulation of gametes and embryos. For male gametes, in vitro manipulation techniques include spermatozoa selection, sorting, immobilization, and quality assessment. A number of studies have been conducted to investigate the application of different laser technologies in the manipulation of human spermatozoa. However, there is a lack of a unified understanding of laser application in the in vitro manipulation of sperm and safety considerations in ART and, subsequently, the inability to make clear and accurate decisions on the clinical value of these laser technologies. This review summarizes the advancements and improvements of laser technologies in the manipulation of human spermatozoa, such as photobiomodulation therapy, laser trap systems for sperm analysis and sorting, laser-assisted selection of immotile sperm and laser-assisted immobilization of sperm prior to ICSI. The safety of those technologies used in ART is also discussed. This review will provide helpful and comprehensive insight into the applications of laser technology in the manipulation of human spermatozoa.

## Background

The term laser is an acronym for light amplification by the stimulated emission of radiation. Since the first laser was developed in 1960, the role that lasers play in various fields, including biology, chemistry, and medicine, has increased steadily [1]. Many procedures have only become possible with the use of lasers. Currently, more than forty different types of lasers have been found in medicine [2]. In the early 1970s, lasers were introduced into the field of gynecology for surgery, excision and ablation of tissue [3–5]. In the 1980 and 1990 s, lasers began to be used in infertility treatment of endometriosis, tubal surgery, ectopic pregnancy and polycystic ovarian syndrome through operative microscopes and laparoscopes [6–8]. Since the successful establishment of assisted reproductive technology (ART), in vitro manipulation of gametes, zygotes and embryos has been an essential integral part of ARTs. Laser technology was introduced into the field of ART as a valuable tool to replace many mechanical and chemical procedures used in the in vitro manipulation of gametes, zygotes and embryos and to optimize the procedural efficiency of techniques such as intracytoplasmic sperm injection (ICSI), assisted hatching, and embryo biopsy [9–11].

For male gametes, in vitro manipulations include techniques for sperm selection, sorting, immobilization and other incubation procedures. The first use of lasers to manipulate human gametes can be traced to 1984 [12]. Sato et al. were the first to report the use of lasers to manipulate human sperm and explore the effects of laser exposure on sperm motility and velocity in vitro [12]. Over the next few years, near-infrared (NIR) laser beams with wavelengths ranging from 700 to 1200 nm were used as optical traps (laser tweezers) in sperm micromanipulation [13–15]. Further development of laser applications was the introduction of an infrared diode laser emitting at a wavelength of 1480 nm, which is far from the absorption peak of DNA (260 nm) [16]. The system allows laser beams along the microscope’s optical axis to the target with minimal absorption by the culture dish and the water molecules [9, 17]. This 1480 nm laser is used across a variety of applications, including sperm selection, sorting, immobilization prior to ICSI, and viability assessment of immotile sperm.

Many studies have been performed to investigate the different laser technologies in in vitro manipulations of human spermatozoa. However, there is a lack of a unified understanding of laser application in the manipulation of spermatozoa and, subsequently, the inability to make clear and accurate decisions on the clinical value of these laser technologies. The present review aims to summarize the advancements and improvements of laser technologies applied in the manipulation of human spermatozoa, such as photobiomodulation, sperm sorting, selection, and immobilization prior to ICSI. We also evaluate the potential value of these laser technologies in the treatment of male infertility and safety considerations for clinical application.

### Photobiomodulation therapy on human spermatozoa

Photobiomodulation (PBM) therapy, previously termed low-level laser therapy (LLLT), generally employs light at red and NIR wavelengths to modulate biological activity [18]. Several studies were conducted in vitro on human spermatozoa through the employment of low-level laser therapy, demonstrating a positive effect on sperm function. PBM parameters have been mostly reported within the red and NIR wavelength range of 600–1100 nm, with an energy density of between 5 and 200 mW/cm^2^. PBM laser devices commonly include Krypton Laser, gallium aluminum arsenide (GaAlAs), neodymium-doped yttrium aluminum garnet, and indium gallium aluminum phosphide (InGaAlP) diode lasers. Table 1 presents a summary of the results.

**Table 1:** Effect of low-level laser therapy (LLLT) on human sperm parameters

Study	Source of sperm	Type	Device	Type of light and wavelength	Intensity/duration of exposure	Main findings and results	References
Sato et al. (1984)	Normal sperm	Fresh	Krypton Laser	Red light (647 nm)	0.5, 1.0, 2.0, 4.0, 8.0, and 32 J/cm^2^/80 and 160 s	Total sperm motility increased after Laser irradiation at 4 J/cm^2^, 8 J/cm^2^, and 32 J/cm^2^ respectively compared with control.	[12]
Lenzi et al. (1989)	Normal sperm	Fresh	LM infrared laser	Infrared laser	5–30 mW/120 s	Laser irradiation had a positive effect on sperm motility.	[22]
Singer et al. (1991)	Normal and abnormal sperm	Fresh	BioBeam instrument	Infrared laser (940 nm)	Maximal intensity 20 mW/cm^2^/4 min	Light exposure significantly increased the percentages of motile, viable, and morphologically normal sperm.	[31]
Firestone et al. (2012)	Normospermia, oligospermia, and asthenospermia	Fresh	Theralaser TLC-1000	Laser light (905 nm)	50 mW/cm^2^/30 s	Low-level laser light had a positive short-term effect on the motility of sperm and did not cause any increase in DNA damage measured at 2 h.	[26]
Salman Yazdi et al. (2014)	Asthenzoospermia	Fresh	GaAlAs laser	GaAlAs laser (830 nm)	4, 6, and 10 J/cm^2^/0, 30, 45, and 60 min	Irradiating human sperms with low-level 830-nm diode lasers can improve their progressive motility depending on both laser density and postexposure time.	[25]
Preece et al. (2017)	Healthy men	Frozen-thawed	Monochromatic coherent laser (Intense 7404)	Red laser light (633 nm)	5.66 mW/cm^2^/35 min; 31mW/cm^2^/30 min	Red light improved sperm motility and did not induce oxidative DNA damage.	[19]
Gabel et al. (2018)	Human	Fresh and frozen-thawed	GaAlAs	GaAlAs single laser (810 nm) and an LED cluster (660 and 850 nm)	GaAlAs single laser: 200 mW/10, 20, and 40 s for frozen sperm, and 15,20, and 300 s for fresh sperm LED cluster: total power 2 W/25, 50, and 75 s for frozen sperm, and 50,100, 200, and 400 s for fresh sperm	The sperm motility index and total functional sperm count increased up to fourfold compared to controls. The motility modification was dependent upon beam irradiance and irradiation time as well as the condition of the sample.	[20]
Highland et al. (2018)	Normal sperm	Fresh	NA	NIR radiation (750–1100 nm)	87 lux/15 min	NIR radiation resulted in a loss of viability and membrane function, increased free radical formation, and induced sperm apoptosis.	[23]
Safian et al. (2020)	Normal sperm	Fresh	Diode laser probes (NILTVIR202 Noura Instruments)	NIR light: 810 nm	0.6 J/cm^2^/NA	PBM treatment before cryopreservation significantly increased the percentages of viable sperm, sperm with high membrane potential, and high mitochondrial activity.	[21]
Safian et al. (2021)	Normal sperm	Fresh	Diode laser (NILTVIR202 Noura Instruments)	Red light (630 nm), NIR (810 nm), or red + NIR (630 + 810 nm)	0.6, 1.2, and 2.4 J/cm^2^/15, 30, and 60 min	The NIR laser at 0.6 J/cm^2^ energy density significantly increased sperm motility and viability and decreased the DNA fragmentation index compared with the red and red + NIR protocols.	[27]
Espey et al. (2022)	Asthenozoospermia and normozoospermia	Fresh	Pulsed laser-probe (Reimers & Jansen)	Pulsed laser-probe (655 nm)	4, 6, and 10 J/cm^2^/0, 30, 60, 90, and 120 min	Exposure to laser energy doses of 4 and 6 J/cm² improved sperm motility and velocity in asthenozoospermic patients.	[24]
Safian et al. 2022	Normal sperm	Fresh	Diode laser (NILTVIR202 Noura Instruments	NIR light: 810 nm	0.6 J/cm^2^/NA	PBM therapy before cryopreservation significantly improved the quality of post-thawed human sperm.	[28]

NA: not available; LED: light emitting diodes; NIR: near-infrared; PBM: photobiomodulation

### Effects of PBM on the parameters of human spermatozoa

#### Sperm motility

Sperm motility is one of the most important characteristics associated with fertility. Almost all studies have examined the impact of PBM therapy on the motility and other kinematic parameters of human sperm. Sato et al. reported that normal sperm samples were exposed to red laser at different dosages, 0.5, 1.0, 2.0, 4.0, 8.0, and 32 J/cm^2^, for 160 s or 80 s and found that total sperm motility significantly increased at 4.0, 8.0 and 32 J/cm^2^ [12]. However, there was no stimulating effect on sperm velocity [12]. A study by Preece et al. assessed the effect of a 633 nm red laser at a power density of 5.66 mW/cm^2^ on frozen human sperm and indicated that the swimming speed improved within 35 min of irradiation [19]. In another work, human fresh and frozen sperm were exposed to light from a GaAlAs single laser beam (810 nm, 200 mW) and an LED cluster (660 and 850 nm, total power 2 W) for different irradiation times [20]. The results showed that the change in sperm motility was dependent upon the stimulatory dose, exposure time, and condition of the sample [20]. Concerning the effects of red and infrared laser irradiation on the motility of sperm, Safian et al. performed three different energy densities of two wavelengths of laser [21]. The results concluded that the NIR laser at 0.6 J/cm^2^ density was superior to the other irradiation protocols in stimulating the effect on motility [21]. The results from the study of Lenzi et al. indicated that the increase in progressive sperm motility after laser irradiation (647 nm) was related to the fast consumption of sperm ATP contents, suggesting that laser irradiation may have an “energetic modulation effect” on normal sperm [22]. However, one study reported by Highland et al. found that NIR irradiation resulted in a damaging effect on sperm viability and a diminished membrane function of sperm [23]. In addition, several studies have used laser beams to stimulate abnormal sperm, including sperm from oligospermia and asthenospermic patients, and showed that irradiation significantly increased sperm motility and velocity [24–26]. In two studies by Safian et al., PBM therapy before human sperm cryopreservation dramatically increased the percentage of live spermatozoa [27, 28].

#### DNA integrity

Sperm DNA integrity is critical for the success of fertilization, embryo development, and implantation and is therefore considered a predictive factor for the clinical outcomes of patients undergoing ART. The potential effect of laser light on sperm DNA integrity has been a focus of attention by investigators. Most published studies have confirmed that red and infrared light does not induce DNA damage. Firestone et al. reported that infrared laser irradiation did not cause any increase in DNA damage at 2 h after exposure in normospermic, oligospermic, and asthenospermic samples [26]. Similarly, exposure to a pulsed-wave laser had no significant effect on the DNA fragmentation level in sperm from asthenozoospermic patients [24]. 830-nm laser irradiation can slightly increase the level of DNA fragmentation in sperm from asthenospermic patients, but it was not statistically significant [25]. The results from the study of Preece et al. showed that red light exposure could not produce sufficiently high levels of reactive oxygen species (ROS) to cause significant oxidative damage in normal sperm DNA [19]. Consistent with previous research, Gabel et al. also found no damage to sperm DNA integrity by light irradiation at a very high-density dose [20]. Interestingly, Safian et al. studied the effects of red and NIR ranges of PBM with a diode laser alone and together on the DNA fragmentation index (DFI) of fresh human sperm and observed that compared with the control, both the red + NIR and red lasers significantly increased DFI, while the NIR range of PBM did not result in a detectable increase in DNA damage [21].

### Mechanism of PBM

Although the underlying pathways of PBM therapy are not well established and may vary among different sperm states (fresh versus frozen, normal versus abnormal), laboratory and clinical studies suggest that PBM significantly improves sperm motility and does not damage DNA. It has become increasingly clear that the biological effects of PBM are closely associated with dosage and irradiation time as well as sperm condition. The lack of consistency in study conditions has always been a confounding factor in the interpretation of PBM mechanisms. Nonetheless, several studies have addressed probable mechanisms regarding the interaction of laser light and spermatozoa.

**Fig. 1:**
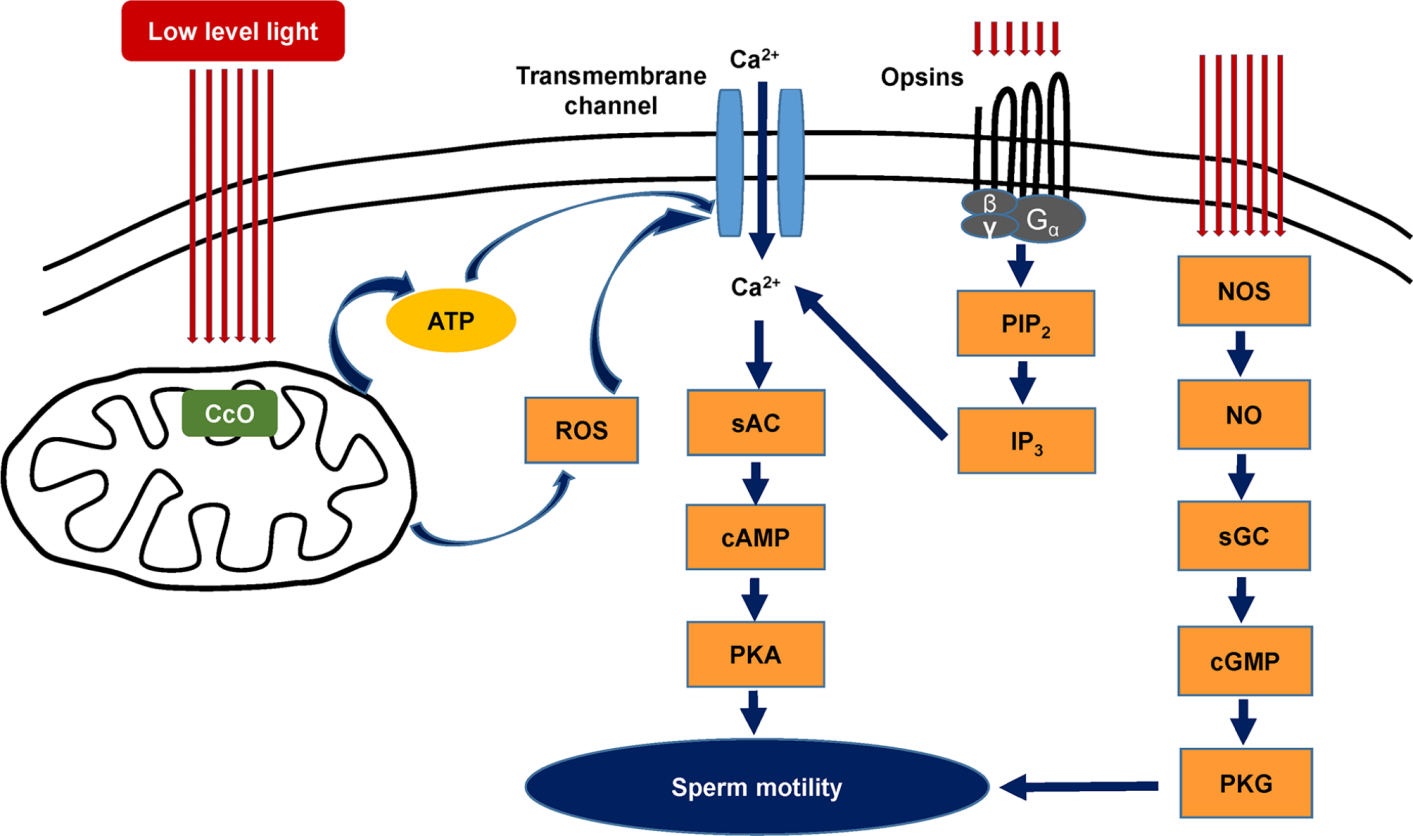
Diagram illustrating four possible mechanisms of PBM therapy on human sperm. Photonic energy in low level light is absorbed by the enzyme cytochrome c oxidase (CcO) located in the mitochondrial respiratory chain. The activated enzyme leads to a proton gradient. Consequently, the levels of reactive oxygen species (ROS) and adenosine triphosphate (ATP) are increased. On the other hand, the application of low level light activates specific receptors of the opsin family coupled to G-proteins in sperm. Phosphatidylinositol 4,5-diphosphate (PIP_2_) is catalyzed by hydrolysis to produce inositol 1,4,5-triphosphate (IP_3_). All of these activities activate light-sensitive ion channels and increase the levels of calcium ions (Ca^2+^). Soluble adenylyl cyclase (sAC) is activated by Ca^2+^. The increased sAC activity activates 3’,5’-cyclic adenosine monophosphate (cAMP)/ protein kinase A (PKA) pathway, thereby promoting sperm motility. In addition, exposure to low level light induces nitric oxide (NO) production by nitric oxide synthase (NOS), which activates cyclic guanosine monophosphate (cGMP)-dependent protein kinases production from soluble guanylate cyclase (sGC). Sperm motility is promoted by activation of protein kinase G (PKG).

At present, at least four mechanisms are believed to be related to the response of sperm to PBM. The first hypothesis suggests that the effects of irradiation are driven by changes in mitochondrial function. Current data indicate that PBM mainly acts on cytochrome c oxidase (CcO) in the mitochondrial respiratory chain [29]. Photonic energy in red or NIR light is absorbed by CcO, resulting in an increased transmembrane proton gradient that drives the production of adenosine triphosphate (ATP), thus increasing sperm motility [19, 20, 30, 31]. ATP is the universal energy source in sperm cells essential for the maintenance of motility driven by flagellar dyneins. An increase in ATP synthesis leads to increased activity of all ATP-driven carriers for ions, such as Na^+^/K^+^ ATPase and Ca^2+^ pumps [32, 33]. Ca^2+^ is a key regulator of sperm motility. Furthermore, since ATP is the substrate of adenylate cyclase, ATP levels control the level of cAMP [34]. Both Ca^2+^ and cAMP are very important second messengers [35, 36]. The second mechanism considers the generation and/or release of reactive oxygen species (ROS) from sperm cells after laser light irradiation. ROS are very small molecules, including oxygen ions, free radicals, and hydrogen peroxide. Previous studies have shown that exposure of sperm to light increases hyperactivated motility mediated by mitochondrial ROS production and the cAMP/PKA pathway [37, 38]. Using the electron paramagnetic resonance (EPR) spin-trapping technique, Lavi et al. followed light-induced hydroxyl radicals in sperm cells and found that the concentration of hydroxyl radicals increased with illumination time and that ROS were produced in both the membrane and cytoplasm [39]. According to a study by Shahar et al., light-stimulated hyperactivated motility was increased through ROS-dependent activation of the epidermal growth factor receptor (EGFR) [38]. The third mechanism for PBM involves nitric oxide (NO), which is an important biological messenger that plays a crucial role in the regulation of energy production and mitochondrial biogenesis [40]. Previous studies revealed that light can induce NO formation by increasing the activity of nitric oxide synthase (NOS) [41–43]. NO is important in sperm motility and capacitation [44, 45]. The fourth hypothesis is linked to the interaction between light and specific receptors of the opsin family, which are coupled to G-proteins in sperm [46]. There is evidence that sperm opsins, specifically rhodopsin, play a role in the response of sperm to light [47]. At least seven opsin proteins are present in human sperm, of which the contents of encephalopsin and neuropsin are the most abundant [48]. The opsins, as light transducers, work through the light activation of photosensitive molecules linked to opsins [47]. The exact function of these opsins in the response of sperm to light still requires further study. Schematic diagrams of four possible mechanisms of PBM therapy on sperm are presented in Fig. 1.

### PBM therapy for male infertility

PBM therapy is a fast-growing technology and provides a promising tool for improving male infertility status [49]. Currently, only a small number of studies have been carried out to evaluate the effect of PBM on the functional capacity of sperm from oligo- and astheno-zoospermia patients [24–26, 50]. Due to a variety of protocol parameters, it is difficult to compare directly between different studies. However, the results of these studies reveal a similar trend; PBM positively affects sperm motility and velocity without causing any damage to the DNA in samples of oligo- and astheno-zoospermia patients [24–26]. In addition, PBM therapy prior to human sperm cryopreservation plays a significant role in improving the quality of postthawed sperm and preventing cryo-damage [21, 28]. However, few studies have conducted assessments of human sperm function, such as acrosome reaction, hyperactivation, and fertilization ability. Recent studies evaluated the effect of PBM therapy on the improvement of spermatogenesis in hyperthermia-induced azoospermia mouse models and found that the spermatogenesis process is significantly improved by PBM therapy [51, 52]. In addition, accumulated evidence from animal studies suggested that PBM therapy improved sperm capacitation and fertilizing ability, as well as reproductive performance [53, 54]. It is postulated that PBM therapy can be considered a promising first-line medical intervention in the treatment of male infertility in the future [30, 31].

Given a lack of guidelines and a limited evidence base for PBM treatment on human sperm, the issues outlined below have been highlighted and addressed for consideration. First, the mechanisms responsible for the beneficial effect on sperm reported by PBM therapy need to be completely elucidated. Theoretically, sperm cells are not exposed to any type of light, so it can be speculated that both normal and abnormal sperm can be sensitive to exogenous bright stimuli. Although different hypotheses have been proposed to explain the effects of light irradiation on sperm, future research is needed to clarify which mechanism plays an important role. Further understanding of the mechanisms is necessary for optimizing clinical treatment. Second, PBM treatment protocols need to be optimized for each type of sperm, such as normal sperm and sperm with mild, moderate, and severe asthenozoospermia. There is now a wide and increasing array of laser equipment to choose from. The effectiveness of PBM therapy is likely to depend on specific laser characteristics. To achieve a desirable clinical outcome, the proper wavelength, pulse duration and energy density must be tailored to the clinical indication. Finally, to evaluate the suitability of PBM for routine clinical use, it is necessary to further study whether PBM has potential genotoxic effects on sperm. DNA integrity is considered a fundamental factor for the fertilization and transmission of paternal genetic information to offspring. Further research on the effects of low-level lasers on sperm cells is imperative.

Taken together, several general conclusions can be drawn: (i) The mechanical basis of PBM therapy is associated with several intracellular metabolism pathways that regulate sperm motility. (ii) The overall results from preclinical and clinical studies suggest that PBM therapy holds promise as a non-invasive treatment for male infertility disorders, such as asthenospermia and oligospermia, by enhancing sperm motility and quality. (iii) PBM beneficial effects depend on wavelengths, exposure time, stimulatory dose, irradiated area, and other treatment parameters (e.g., condition of the sample). Tailoring PBM treatment protocols for specific sperm conditions is essential.

### Micromanipulation of human sperm using a laser optical trap

An optical trap is a noninvasive biophotonic tool that has been studied for practical applications in a variety of fields, including physics, chemistry, biology, and medical science [55, 56]. A laser optical trap was first reported to manipulate single cells in 1987 [57]. The authors achieved damage-free trapping and manipulation of suspensions of single cells and organelles located within individual living cells [57]. Berns et al. first showed that a laser trap could be used to move the chromosomes inside mitotic cells in vitro [58]. Not long afterward, single-point laser traps were used as a tool to manipulate individual sperm cells and analyze the interaction between laser and sperm and sperm motility by measuring sperm swimming forces in the late 1980s [13, 59]. Several studies have demonstrated that laser optical trapping and micromanipulation of sperm cells using a NIR beam is technically feasible [14, 15, 60–70].

### Laser trapping optical system

The optical trapping system is designed as a biomedical tool to study the physiological and biomechanical properties of cells. This system introduces NIR laser light into an inverted microscope, creating a single-point and three-dimensional gradient laser trap at the focal point of the microscope. Trapping provides a noninvasive method for analyzing and classifying sperm based on sperm swimming speed and swimming force [13–15, 60, 65–67, 70] and studying the effects of light radiation [63], oxidative phosphorylation inhibitors [64], freezing [62], drugs [64], and other factors [61, 68, 69].

**Fig. 2:**
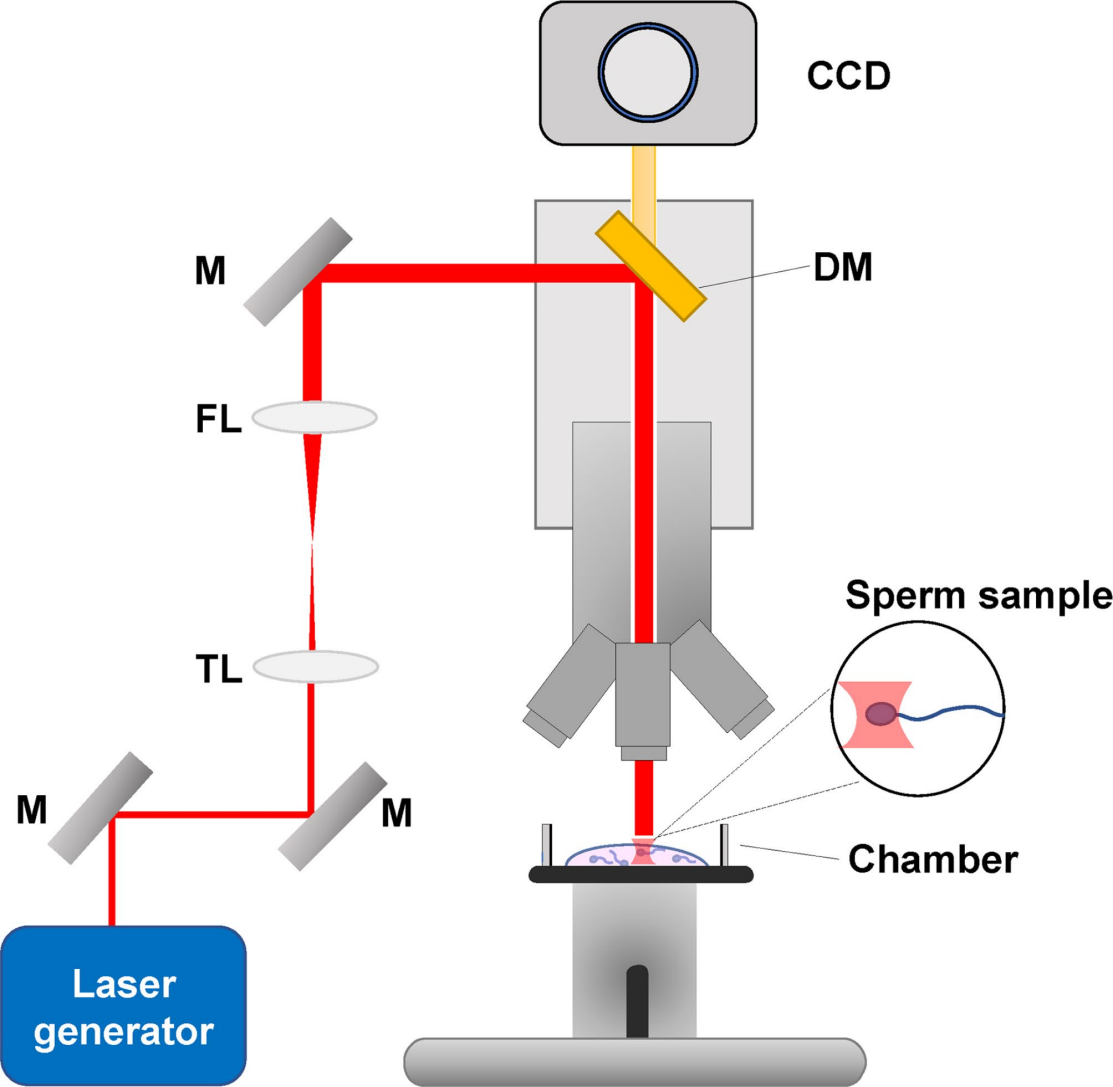
Schematic diagram of the laser system for sperm trapping. M: mirrors; TL: telescope lens; FL: focusing lens; DM: dichroic mirror; CCD: charge-coupled device

The laser-induced optical trap reported in the literature consisted of a laser operating in a continuous wave at a wavelength of 760–1070 nm, which traveled through a series of mirrors and lenses into the microscope, as shown in Fig. 2. Laser devices generally included the neodymium:yttrium-aluminum-garnet (Nd:YAG) laser, titanium-sapphire laser, and CW ytterbium fiber laser. In the described automatic microscope system, real-time tracking and trapping were described, which provided a user-friendly robotic interface. Sperm cells were continuously tracked, and the changes in sperm swimming behavior were dynamically monitored. To improve the accuracy of sperm analysis and sorting, automatic annular laser trapping was developed [67]. Table 2 presents a summary of the laser traps used for sperm analysis and sorting.

**Table 2:** Summary of laser traps (optical tweezers) used for sperm analysis and sorting

Study	Source of sperm	Type	Type of laser	Wavelength	Trapping power	Exposure time	Total number of sperm trapped	Analysis parameters	References
Tadir et al., 1989	Donor	Fresh	Neodymium: Yttrium-Aluminum-Garnet (Nd:YAG) laser	1.06 μm	1 W	15, 30, 45, 60, 90, and 120 s	514	Linear velocity, actual distance traveled, maximum lateral head displacement and motility patterns (before, during, and after exposure)	[13]
Tadir et al., 1990	Donor/normal	Fresh	Neodymium: Yttrium-Aluminum- Garnet (Nd:YAG) laser	1.06 μm	10–150 mW	< 10 s	705	Linear velocity, motility patterns	[14]
Colon et al., 1992	Donor/normal	Fresh	Neodymium-doped yttrium aluminum garnet (Nd:YAG) laser	1.06 μm	25–96 mW	30, 60, 120, and 180 s	NA	Sperm velocity	[60]
Westphal et al., 1993	Donor/normal	Fresh	Titanium-sapphire laser	760 nm	< 300 mW	< 10 s	892	Linear motility, hyperactivated motility, and cumulus-related motility	[61]
Dantas et al., 1995	Normal	Fresh	Titanium-sapphire laser	800 nm	0-300 mW	NA	2130	Relative escape force of sperm	[62]
Liu et al., 1996	Donor/normal	Fresh	Nd:YAG laser	1064 nm	< 500 mW	< 10 min	NA	Sperm cell temperature, DNA structure, viability, and pH	[15]
König et al., 1996	Donor/normal	Fresh	Tuneable cw Ti: Sapphire ring laser,	UVA: 320–400 nm; NIR: 760 nm, 800 nm	1.5 mW (UVA), 105 mW (NIR)	2 min	580	Cytotoxic effect	[63]
Patrizio et al., 2000	Normozoospermic and asthenozoospermic donors	Fresh	Nd:YAG laser	1064 nm	< 500 mW	NA	≥ 80 per patient	Relative escape force of sperm	[64]
Shao et al., 2007	Donor	Frozen	CW Ytterbium fiber laser	1070 nm	0–25 mW	NA	93	Curvilinear velocity , smooth path velocity, amplitude of lateral head displacement	[65]
Nascimento et al., 2008	Donor	Fresh and frozen	NA	1064 nm	NA	NA	NA	Curvilinear velocity, escape force	[66]
Shi et al., 2009	Donor	Frozen	CW Ytterbium fiber laser	1070 nm	< 24 mW	NA	264	Curvilinear velocity, smooth path velocity, amplitude of lateral head displacement	[67]
Hyun et al., 2012	Normal	Frozen	Nd:YVO_4_ laser	1064 nm	450 mW	NA	NA	Curvilinear velocity, minimum laser power	[68]
Auka et al., 2019	Donor	Frozen	CrystaLaser	1064 nm	700 mW	NA	479	DNA analysis	[69]
Zhong et al., 2022	Infertility patients	Fresh	Fiber laser	NA	~ 400 mW	< 20 s	NA	Longitudinal rolling dynamics of single sperm	[70]

NA: not available; NIR: near-infrared; UVA: ultraviolet A

### Effects of laser trapping on sperm quality

Early studies on sperm quality parameters using the force generated by infrared laser beams mainly focused on the power and swimming force loaded onto the trapped sperm when it escaped out of the optical trap. Single-spot, gradient-force laser tweezers were first used to trap human sperm cells to study velocity in the late 1980s [13]. Sperm velocity was not significantly affected by the trap after short periods of exposure, but prolonged exposure time led to a decrease in sperm velocity [13]. Tadir and coworkers later found that there existed a positive correlation between sperm velocity and laser power, and the average trapping power varied depending on sperm linear velocities [14]. Based on previous studies, Colon et al. developed a three-dimensional laser optical trap to explore the effect of manipulation time on sperm velocity [60]. The results from Liu et al. demonstrated that it was feasible to monitor sperm cell physiology in situ during continuous wave and pulsed laser trapping [15]. In addition, the authors also found that optical tweezers and microbeams interfered with the levels of cell metabolic activity and sperm viability [15]. Although single-point laser traps provided a way to assess the motility and quality of individual sperm, they also had some drawbacks, among which the biggest problem was low throughput. Soon thereafter, a 3-D annular laser trap was developed and applied to study many aspects of sperm cellular physiology [66] and provided the possibility of multilevel, high-throughput sorting and analysis of human sperm based on motility [65, 67]. Recently, Zhong et al. used an optical trap to determine the chirality and frequency of longitudinal rolling of human sperm, which was expected to achieve automatic evaluation and separation of single sperm in the future [70].

### Optical traps as a tool for studying the impact of the external environment on sperm motility

The motility generated by sperm swimming is attributed to flagellar movement, which is not only a fundamental expression of sperm viability but also essential for evaluating fertilization ability. Because the change in momentum of the photons produces a force acting on an object, optical traps can confine and manipulate sperm cells. Sperm motility was studied by Tadir et al. using force generated by a laser-generated optical trap [13]. The swimming force of sperm is qualified by measuring the minimum trapping force required to keep sperm in traps, which is proportional to the applied laser power. This value is also defined as the relative escape force.

In a study by Westphal et al., a laser optical trap was used to investigate the relative force generated by human sperm displaying different motility patterns [61]. The results showed that the relative force was associated with the motility patterns (linear, hyperactivated, and cumulus-related), and exposure of sperm to the cumulus mass resulted in the greatest relative force [61]. Araujo et al. used a laser trap to compare the relative escape force of human epididymal sperm with that of human ejaculated sperm and concluded that the average relative escape force of epididymal sperm was 60% weaker than that of ejaculated sperm [71]. Dantas et al. studied the effect of freezing on sperm escape force and found that there was no significant difference in the overall mean relative escape forces between fresh and frozen-thawed sperm [62]. Patrizio et al. investigated the effect of pentoxifylline on the swimming forces of human sperm and demonstrated that pentoxifylline significantly increased sperm intrinsic relative force in sperm from normozoospermic and asthenozoospermic samples [64]. Hyun et al. reported the effect of media with different viscosities on sperm motility and found that the sperm swimming force increased with increasing viscosity [68]. Chow et al. found that 633 nm red light irradiation significantly increased the mean squared displacement, which is related to an increase in the swimming force [72].

In total, it can be concluded that 3-D annular laser traps have more advantages than single-beam traps for high-throughput sorting and analysis of sperm quality, including motility, swimming velocity, and force. Moreover, optical traps, as a noninvasive tool, may also play a unique role in assessing the impact of the external environment on sperm motility.

### Laser-assisted selection of viable but immotile sperm

Viable sperm are necessary for successful ICSI. Generally, sperm motility is the main sign used to determine sperm viability. However, the challenge faced by embryologists is how to judge whether the sperm is dead or viable when encountering sperm samples without obvious vitality. The absence of motile spermatozoa is often seen in absolute asthenozoospermia samples, epididymal sperm aspirations and testicular biopsy specimens [73, 74]. It is necessary to establish a fast and simple method to distinguish between dead and viable but immotile sperm. Several testing methods have been developed to select viable but immotile sperm for ICSI, including a hypo-osmotic swelling test (HOST), chemical substances for induction of tail movements, mechanical touch technique and laser-assisted immotile sperm selection [75–79]. Table 3 shows the advantages and disadvantages of different immotile sperm selection techniques.

**Table 3:** Advantages and disadvantages of immotile sperm selection techniques

Procedures	Principle	Advantage	Disadvantage	References
Hypo-osmotic swelling test (HOST)	HOST is based on the semi-permeability of intact cell membranes. The tail of viable sperm curves or swells after exposure to a hypo-osmotic medium.	• Simple and economical • Repeatable and reliable • Recommended in the WHO laboratory manual	• Need for prior treatment • Time-consuming • Not suitable for cryopreserved sperm	[75–79]
Pharmacological stimulation	The mechanism of drug-induced motility is mainly to activate signaling pathways related to sperm motility.	• Relatively easy to use • Easy identification of viable sperm	• Need for pharmacological agents • Need for prior treatment • Possible side effects	[111–113]
Mechanical testing	The ICSI pipette is used to test the elasticity of the sperm tail. If sperm exhibiting elastic tails is presumed to be viable.	• Simple and economical • No need for additional reagents and equipments	• Highly dependent on practical experiences • Not easy to identify viable sperm • A relatively small number of relevant literatures	[73, 114]
Laser-assisted selection	The end of the sperm tail was targeted with a laser pulse. Those sperm that presented with curling of the tails were regarded as viable.	• Time-saving • Easy to perform • Noncontact • Can be performed during ICSI	• Need for laser equipment • Still not standardized	[79, 80, 83, 84, 86–89]

WHO: World Health Organization; ICSI: intracytoplasmic sperm injection

The introduction of laser-assisted immotile sperm selection is an advancement in the use of lasers in the treatment of male infertility. The laser can help discriminate between viable sperm and dead sperm in cases of completely immotile sperm. The mechanism may be related to sperm membrane integrity [80]. Viable sperm treated by laser exhibit tail curling due to the instantaneous opening of the membrane. Dead sperm show no reaction, as the membrane integrity has been undermined. Laser-assisted selection is superior and most suitable for routine application in ART laboratories compared with the other testing methods [79]. The main advantage of this method is that it is convenient and reliable [81, 82]. It does not require chemical compounds to induce sperm motility, so it does not produce side effects. Most importantly, this technique is suitable for the different types of immotile sperm [79]. Frozen sperm spontaneously develop tail swelling, which may bias the results. Laser-assisted selection is especially used for sperm that have been frozen and then thawed. The main results are reported in Table 4.

**Table 4:** Clinical applications of diode lasers in the selection of immotile but viable spermatozoa before ICSI

Study	Source of spermatozoa	Type	Intensity of laser	duration of exposure	No. cycles of ART	No. oocytes fertilized/Total oocytes	No. pregnancies	No. live births	References
Aktan et al. (2004)	24 patients with complete asthenozoospermia (complete immotile spermatozoa) and 21 patients with either obstructive or nonobstructive azoospermia (testicular spermatozoa)	Fresh	120 µJ	1.2 ms	24 (ejaculate) 21 (testicular)	64.2% (NA) 45.4% (NA)	33.3%(8/24) 25.0%(5/20)	28.0%(7/25) 19.0%(4/21)	[80]
Gerber et al. (2008)	A patient with Primary cilia dyskinesia	Fresh	400 µJ	5 ms	1	57.1% (4/7)	1	NA	[83]
Nordhoff et al. (2013)	48 patients with testicular biopsy	Fresh	NA	6-7ms	58 TESE-ICSI cycles 65 control cycles	52.7% (292/554) 42.1% (216/617)	NA	NA	[85]
Chen et al. (2015)	Two patients with obstructive azoospermia and severe asthenospermia	Fresh	NA	2 ms	2	83.3%(10/12); 100%(6/6)	2	NA	[86]
Chen et al. (2017a)	A patient with nonobstructive azoospermia (testicular spermatozoa)	Frozen-thawed	200 µJ	2 ms	1	80% (4/5)	1	NA	[87]
Chen et al. (2017b)	7 patients with severe asthenozoospermia (complete immotile spermatozoa) and 25 patients with azoospermia (testicular spermatozoa)	Fresh and frozen-thawed	120 µJ	1.2 ms	NS	78.7% (37/47) (ejaculate) 80.3% (139/173)(testicular)	NA	NA	[88]
Ozkavukcu et al. (2018)	A patient with Kartagener’s syndrome	Fresh	NA	350 µs	1	45.5% (10/22)	1	1	[84]
Chen et al. (2021)	33 TESA-ICSI cycles/99 controls	Fresh and frozen-thawed	200 µJ	2 ms	33 (test group) 99 (control group)	78.2%(283/362) 80.5% (763/948)	61.2% (30/49) 47.5% (76/160)	69.7% (23/33) 60.6% (60/99)	[89]

NA: not available; TESA: testicular aspiration; ICSI: intracytoplasmic sperm injection

The use of lasers for the selection of viable but immotile sperm was first introduced by Aktan et al., who performed a direct laser shot of 129 µJ for approximately 1.2 ms to the tip of the sperm tail [80]. Sperm that showed curling or coiling of the tail were regarded as viable. Since nonviable sperm had no such change, this method helped to identify sperm with membrane functional integrity [80]. Laser selection of sperm from ejaculated absolute asthenozoospermic samples and testicular biopsies showed a similar percentage of viable sperm compared with the hypo-osmotic swelling test. Gerber et al. and Ozkavukcu et al. separately reported a case with primary cilia dyskinesia (PCD) who achieved a normal pregnancy and live birth by laser-assisted viability assessment of immobile sperm during ICSI [83, 84]. In a large sample study, Nordhoff et al. compared sperm selection with light microscopy with laser-assisted selection and found that the fertilization rate improved significantly when applying laser selection [85]. Chen et al. reported that two successful pregnancies were achieved by using laser selection of viable but immotile sperm from two cases of obstructive azoospermia and severe asthenospermia [86]. The team of Chen et al. first reported a successful pregnancy using completely immotile frozen-thawed testicular spermatozoa selected by laser [87]. The team of Chen et al. continued to report the results of cryopreservation of completely immotile testicular sperm or ejaculate sperm of 32 patients by laser-assisted selection and showed similar rates of fertilization, cleavage and good-quality embryos compared to routine fresh immotile sperm [88]. This study confirmed that combined with laser-assisted selection of viable sperm, completely immotile sperm can be frozen for fertility preservation [88]. Recently, a retrospective comparative study by Chen et al. demonstrated that no negative effect on perinatal and neonatal outcomes was observed in patients with immotile sperm by using laser-assisted selection for ICSI [89].

In summary, laser-assisted immotile sperm selection, an innovative technique used in ART, is simple and effective and does not require the use of additional substances to either induce motility or cause sperm flagellum curling, consequently there is no accompanying damage to sperm or embryos. Furthermore, several clinical studies have shown promising results in male infertility treatment and fertility preservation, with a focus on safety and positive reproductive outcomes.

### Laser-assisted immobilization of sperm

Sperm immobilization prior to ICSI is necessary for successful fertilization to occur [90, 91]. Mechanical immobilization is achieved by pressing the sperm tail to the bottom of a culture dish using the tip of an ICSI injection needle [92–96]. This method has been regarded as a classic method for many years. However, an alternative method of sperm immobilization was described in which a noncontact diode laser system was used [97–104]. Immobilization of sperm was performed by a single or double laser shot [97–100, 102–104]. Table 5 shows the advantages and disadvantages between laser-assisted and mechanical immobilized sperm. Laser-assisted immobilization has two advantages: first, it avoids the negative impact of polyvinylpyrrolidone (PVP) on sperm during mechanical immobilization; second, it simplifies the operation process and shortens the manipulation time. Sperm immobilization with this method was found to yield comparable results in the rates of fertilization [98–100, 104], cleavage [98, 104], and good-quality embryos [100, 104] compared with mechanical immobilization. Table 6 presents a summary of laser-assisted immobilization of sperm.

**Table 5:** Comparison of advantages and disadvantages between laser-assisted and mechanical immobilized sperm

Procedures	Principle	Advantage	Disadvantage	References
Mechanical immobilization	Immobilization is performed by touching the tail of the sperm with ICSI pipette.	• No need for additional equipment • Easy to standardize and adapt in clinical scenarios	• Resulting in sperm membrane damages • Require PVP to facilitate sperm capture	[92–97]
Laser-assisted immobilization	Sperm is immobilized with 1.48 μm wavelength diode laser.	• Simple and time-saving • Retaining membrane integrity • Noncontact • Avoiding the harmful effects of PVP on sperm and embryos	• Need for laser equipment • Literature insufficiency on the percentage of live birth	[79, 97–99, 101, 102]

ICSI: intracytoplasmic sperm injection; PVP: polyvinylpyrrolidone

**Table 6:** Summary of laser-assisted immobilization of sperm for intracytoplasmic sperm injection

Study	Source of sperm	Strategy for immobilization of sperm	Target regions for immobilization	Intensity of laser	Duration of exposure	No. oocytes fertilized/Total MII oocytes)	No. good-quality embryos/Total MII oocytes	No. pregnancies	No. live births	Main findings	References
Montag et al. (2000)	Fresh ejaculated sperm	Single or double laser shot	The middle region of the sperm tail	0.25 mJ, 0.5 mJ, 1.0 mJ, 2.0 mJ, 2.5 mJ, 3.0 mJ	NA	NA	NA	NA	NA	Laser can be used for immobilization and membrane permeabilization of the sperm tail.	[102]
Ebner et al. (2001)	Fresh ejaculated sperm	Double laser shot	The middle and end regions of the sperm tail	1.5 mJ, 1.0 mJ	15 ms, 10 ms	174/262 (66.4%)	58/262 (22.1%)	NA	NA	There were no significant differences in rates of fertilization, early cleavage, or blastocyst formation between laser-assisted immobilization and mechanical immobilization groups.	[100]
Eroglu et al. 2002	Fresh ejaculated sperm	NA	The middle region of the sperm tail	NA	NA	13/78 (17%)	NA	NA	NA	Laser-assisted immobilization of sperm may be useful in ICSI procedures.	[101]
Ebner et al. (2002)	Fresh ejaculated sperm	Double laser shot irradiations	The middle and end regions of the sperm tail	1.5 mJ, 1.0 mJ	15 ms, 10 ms	3/4 (75%)	NA	1	1	Successful birth was achieved after transfer of embryos derived from laser-immobilized sperm.	[99]
Debrock et al. (2003)	Fresh ejaculated sperm	Double laser shot	Aside of the middle of the sperm tail followed by a second shot directly to the middle of the sperm tail	NA	NA	89/179 (49.7%)	NA	3/10 (30%)	2/10 (20%)	No difference was observed in fertilization rate, cleavage, or blastocyst formation when oocytes were injected with sperm immobilized with laser compared to sperm immobilized with mechanical method.	[98]
Li et al. (2004)	Fresh sperm from testicular biopsies	Single laser shot	Behind the middle section of the sperm tail	NA	1 ms	68/95 (71.6%)	51/95 (53.7%)	NA	NA	There were no significant differences in rates of fertilization, cleavage, and good-quality embryos between laser immobilization and mechanical immobilization groups.	[104]
Xu et al. (2007)	Fresh ejaculated sperm	Single laser shot	Behind the middle section of the sperm tail	NA	0.45 ms	NA	NA	NA	NA	Laser-assisted sperm immobilization did not cause direct damage to the sperm DNA.	[103]
Chan et al. (2017)	Fresh ejaculated sperm	NA	Head and midpiece region; principal piece Region; end piece region	NA	NA	NA	NA	NA	NA	Laser-assisted immobilization did not result in any external membrane damages besides morphological changes at SEM level.	[97]

NA: not available; SEM: scanning electronic microscopy; ICSI: intracytoplasmic sperm injection

The first study of 1.48 μm wavelength laser-assisted immobilization of human sperm for ICSI was reported in the early 2000s. Montag et al. evaluated two strategies for the immobilization of human sperm using a laser: a single laser shot and a double shot. After injecting human laser-assisted immobilized sperm, mouse oocytes were successfully activated and formed pronuclei [102]. A prospective self-controlled study by Ebner et al. found no significant differences in the rates of fertilization, early cleavage, or blastocyst formation between laser-assisted immobilization and mechanical immobilization groups [100]. These results were confirmed by the data from two studies by Debrock et al. [98] and Li et al. [104] reporting no difference in fertilization rate, cleavage, or blastocyst formation of oocytes injected with sperm that were immobilized with laser compared to sperm immobilized with the mechanical method. Ebner et al. first reported a live birth achieved with sperm that were immobilized by using two successive laser shots [99]. Xu et al. studied the effect of laser-assisted immobilization on sperm DNA and demonstrated that sperm immobilization by laser did not cause direct damage to the sperm DNA [103]. Chan DYL and Li TC explored the impact of the different methods of immobilization on the external morphology and function of sperm at the scanning electronic microscopy (SEM) level [97]. The results showed that laser-assisted immobilization did not lead to any observable external membrane damage besides distinctive morphological changes at the SEM level.

In short, laser-assisted immobilization of sperm prior to ICSI is a potentially useful alternative to the conventional mechanical method. Laser-assisted immobilization is effective in achieving successful fertilization and does not harm sperm DNA. More research is needed to focus on the relevance and safety of clinical outcomes.

### Safety considerations for clinical application

In the past 3 decades, technological advances and the use of lasers in the assisted reproductive technology laboratory have developed rapidly. Safety has always been an important aspect of any laser application in the in vitro manipulation of human sperm. Most concerns focus on the effects on sperm DNA integrity, a basic factor for fertilization and transmission of genomic information from the father to the offspring [105, 106].

The wavelength, pulse duration, and energy density are all factors that must be taken into consideration when evaluating the safety of lasers [10, 107]. The emitted wavelength of a laser usually covers the entire light spectrum from infrared to ultraviolet (UV). UV lasers have been reported to induce potential genetic damage to gametes, which is believed to be the result of UV photoabsorption by DNA and RNA, as well as photooxidation reactions [63, 108]. Consequently, most lasers that are used in the ART laboratory generate light within the visible (400–760 nm) and NIR (760-1,400 nm) ranges. To minimize the damage caused by thermal effects, the ideal pulse duration should be lower than the thermal relaxation time of the target chromophore [109]. The pulse durations employed in ART vary from 20 ms down to the femtosecond level [109]. The appropriate energy density, delivered by the laser beam, needs to be determined based on their intended use.

For PBM therapy on spermatozoa, low-level laser light is applied to trigger sperm cell biostimulation, which leads to photochemical and photophysical reactions and avoids heating or thermal effects [110]. Several studies have demonstrated that low-level red light exposure does not induce a statistically significant effect on DNA damage [19–21, 24–26, 28]. In contrast, investigating the sensitivity of human sperm to NIR radiation showed that exposure to NIR for 15 min severely damaged sperm membrane permeability and function and led to reduced sperm viability [23]. The authors found significant increases in the percentages of disrupted DNA-protamine toroid assembly and sperm with chromatin dispersion after exposure to NIR, suggesting a profound detrimental effect on DNA integrity when exposed to NIR radiation [23]. Research on PBM therapy on human sperm has made significant progress, but there is still a distance from clinical application. Further studies will be necessary to exclude potential genotoxic effects on sperm and to clarify the suitability of PBM therapy for clinical application.

A further expansion in laser-assisted immobilization of sperm was the development of a technique for the use of lasers for the selection of viable but immotile sperm. Both energy levels of the laser pulse are slightly different. A single shot of 120–400 µJ is sufficient to detect viable but immotile sperm [80, 83, 87–89]. For the immobilization of sperm using a double shot strategy, due to splitting up energy into two separate pulses, the total energy applied to the sperm should be higher compared to the selection of immotile sperm. However, both applications of laser shots are aimed near the end of the sperm, which is far from the sperm head containing the genetic material. Xu et al. did not find any direct damage to sperm DNA when a laser was applied to immobilize sperm, even though the author used two methods to detect sperm DNA damage [103]. A study by Chan DYL and Li TC showed that laser-assisted immobilization did not result in any external membrane damage at the scanning electronic microscopy level [97].

To summarize, there are now increasingly more available laser technologies for the in vitro manipulation of human sperm. In all cases described thus far, the application of laser technologies has been proven to be useful and effective. Proper laser selection for indication and treatment requires a deep understanding of the laser principles and sperm structure and function. This will help to reduce the risk of damage to sperm DNA and allow researchers to obtain better results.

## Conclusions

The laser system is now being applied across ART laboratories and provides a promising tool for the diagnosis and treatment of subfertility and male factor infertility. Some of the laser technologies described in this review are already mature and routinely applied in daily work. Studies have confirmed that laser-assisted operation in the selection of immotile sperm and sperm immobilization did not appear to increase the risk of adverse neonatal outcomes. However, some laser technologies, such as laser tweezer and PBM therapy, have still only had proof-of-principle demonstrations. The research data are not yet abundant. Although the use of lasers in the manipulation of human sperm offers several benefits. However, there are several drawbacks and challenges that will be faced.

Standardization is lacking. In laser-based sperm manipulation research, laser parameters should be united including wavelength, energy density, and exposure time. Standardized protocols and methodologies are needed for various sperm conditions, along with safety assessments. Standardized reporting guidelines should be adopted for detailing laser parameters, samples, and outcomes. Without standardization, comparison of results and determination of optimal settings may be hindered. Therefore, developing standardization for laser-based sperm manipulation should be a priority in future research efforts to enhance consistency.

Safety assessment is limited. Considering the potential damaging effects of different laser procedures and treatments described in this review on sperm DNA integrity, many critical treatment phases may potentially affect epigenetic, genetic, or chromosomal errors during embryo development. Thus, the ideal paradigm for introducing a new approach would be first to experiment on animals and then research should be conducted on donated human gametes for appropriate risk assessment. Additionally, sufficiently powered randomized controlled trials (RCTs) need to demonstrate the clinical benefit and safety of the different laser approaches, where appropriate, follow-up of newborns.

This review provides current knowledge on some laser techniques applied in the manipulation of human sperm. Undoubtedly, this field seems very promising because the techniques are new and beneficial. Meanwhile, there are still some challenges that need to be faced because the technologies encompass sophisticated equipment and complex experimental procedures. Therefore, a deeper comprehension of the mechanism of laser action on sperm cells is required for the optimization of the protocols in clinical practice, and the refinements based on evidence-based medicine are also a priority for future research.

## Data Availability

All data generated or analyzed during this study are included in this published article.

## Abbreviations

ART: Assisted reproductive technology
ICSI: Intracytoplasmic sperm injection
PGT: Preimplantation genetic testing
PBM: Photobiomodulation
LLLT: Low-level laser therapy
NIR: Near-infrared
GaAlAs: Gallium aluminum arsenide
InGaAlP: Indium gallium aluminum phosphide
DFI: DNA fragmentation index
CcO: Cytochrome c oxidase
ROS: Reactive oxygen species
EPR: Electron paramagnetic resonance
EGFR: Epidermal growth factor receptor
NO: Nitric oxide
NOS: Nitric oxide synthase
Nd:YAG: Neodymium: Yttrium-Aluminum-Garnet
PCD: Primary cilia dyskinesia
PVP: Polyvinylpyrrolidone
SEM: Scanning electronic microscopy
UV: Ultraviolet
WHO: World Health Organization
HOST: Hypo-osmotic swelling test
